# Comparison of retention in observational cohorts and nested simulated HIV vaccine efficacy trials in the key populations in Uganda

**DOI:** 10.1186/s12874-020-00920-4

**Published:** 2020-02-12

**Authors:** Andrew Abaasa, Jim Todd, Stephen Nash, Yunia Mayanja, Pontiano Kaleebu, Patricia E. Fast, Matt Price

**Affiliations:** 1 MRC/UVRI & LSHTM Uganda Research Unit, Entebbe, Uganda; 2 London School of Hygiene and Tropical Medicine, London, UK; 3 International AIDS Vaccine Initiative, New York, USA; 4 Pediatric Infectious Diseases, School of Medicine, Stanford University, Palo Alto, California, USA; 5 Department of Epidemiology and Biostatistics, University of California at San Francisco, San Francisco, USA

**Keywords:** Retention dropout observational cohorts vaccine trials key-populations

## Abstract

**Background:**

Outcomes in observational studies may not best estimate those expected in the HIV vaccine efficacy trials. We compared retention in Simulated HIV Vaccine Efficacy Trials (SiVETs) and observational cohorts drawn from two key populations in Uganda.

**Methods:**

Two SiVETs were nested within two observational cohorts, one in Fisherfolk (FF) and another one in Female Sex Workers (FSW). Adult participants in each observational cohort were screened for enrolment into SiVETs. Those screened-out or not screened continued participation in the observational (non-SiVET) cohorts. SiVET participants were administered a licensed hepatitis B vaccine in a schedule that mimicked an actual HIV vaccine efficacy trial. Both cohorts were followed for 12 months and retention was assessed through dropout, defined as lost to follow up, being uncontactable, refusal to continue or missing the last study clinic visit. Dropout rates were compared using Poisson models giving rate ratios and 95% confidence intervals (95%CI).

**Results:**

Out of 1525 participants (565 FF and 960 FSW), 572 (38%) were enrolled into SiVETs (282-FF and 290-FSW), and 953 (62%) remained in the non-SiVET cohorts. Overall, 326 (101 SiVET, 225 non-SiVET) dropped out in 1260 Person Years of Observation (PYO), a dropout rate of 25.9 /100 PYO (95%CI: 23.2–28.8); fewer dropped out in the SiVET cohorts (18.4, 95% CI: 15.1–22.4) than in the non-SiVET cohorts (31.6, 95% CI: 27.8–36.1), rate ratio (RR) =0.58, 95% CI: 0.46–0.73. In all cohorts, the dropout was more marked in FSW than in FF population. Duration lived in community was associated with dropout in both SiVETs and religion in both non-SiVET cohorts.

**Conclusion:**

The rate of dropout was lower in SiVET compared to non-SiVET cohort. Though the difference in dropout between SiVET and non-SiVET was generally similar, the actual dropout rates were higher in the FSW population. Conduct of SiVETs in these key populations could mean that designing HIV Vaccine Efficacy Trials will benefit from lower dropout rate shown in SiVET than non-SiVET observational cohort.

## Background

Populations with high HIV incidence and good retention in follow up are needed to ensure successful conduct of efficacy trials of the HIV vaccines being developed [1]. In countries, where the general population HIV incidence is low [2, 3] to be used for this purpose, sub populations such as key populations could be a better alternative. However, key populations are perceived to be highly unstable and difficult to keep in follow up [4–7]. Observational cohorts in key populations in Africa have shown high HIV incidence [8–13] but slightly lower study completion (70–76%) [9, 13–15] than in the general population (85%) [3]. Attrition from studies could bias the estimate of outcome measures and diminish statistical power. Estimation of expected trial attrition is an important component of trial planning to avoid the risk of type II error or higher expense and unethical concern of following up more than the necessary number of trial participants.

Contrary to perception that Fisherfolks (FF) on the shoreline of Lake Victoria and Female sex workers (FSW) in Kampala, Uganda are highly mobile populations and hard to maintain in follow up [6, 16], these populations could be suitable for HIV vaccine efficacy trials. Studies in these key populations have demonstrated very high HIV incidence of 3 to 11 per 100 person years [8, 9, 11, 12, 14, 15], willingness to participate > 90% [17, 18] and relatively good retention in follow up > 75% [6, 9, 19, 20].

To date, no HIV efficacy trials have completed follow up in these populations and the available information comes from observational cohorts. Studies have shown how differences in the selection of participants into a trial affects HIV incidence compared to observational cohorts in the same populations [8, 21–23] but we do not know how the dropout rate would compare under similar settings. During the conduct of efficacy trials, participants are seen more regularly and techniques such as phone call reminders and home visits are employed to keep participants in follow up. This level of attention is likely to be higher than that in observational cohorts and could improve adherence to clinic attendance as well as completion of trial procedures beyond what is seen in observational cohorts. Therefore, planning HIV vaccine efficacy trials in key populations assuming the same dropout rate seen in observational data could be misleading. Inaccurate information on dropout rates predicted at trial planning stage could result in an over or under estimate of the study sample size, resulting in either unnecessary cost or diminished statistical power for the outcome. Accurate information on attrition in the FF and FSW populations is needed to inform the design of HIV vaccine efficacy trials in these and similar populations. In this paper we use data from two Simulated Vaccine Efficacy Trials (SiVETs) nested within observational cohorts in the FF and FSW populations in Uganda to (i) compare the dropout rates in the SiVET (interventional) cohorts to that in the non-SiVET (observational) cohorts, (ii) report reasons for dropout and (iii) determine factors associated with dropout.

## Methods

### Design and setting

The data used in this paper were obtained from two observational cohorts. Observational cohort one (OBC_1_) was in the FF population, from January 2012 to April 2015 at MRC/UVRI and LSHTM Uganda Research Unit clinic located in Masaka town about 100 km West of Kampala, the capital of Uganda. OBC_1_ recruited from fishing communities located on the shoreline of Lake Victoria in Masaka district. Houses in the fishing communities are mainly made of wattle-and-mud or iron sheeting, and concentrated on the edge of swamps. While the main economic activity is fishing, there are small-scale businesses and services supporting the fishing occupation and the cohort was recruited from all occupations.

The second observational cohort (OBC_2_) was in the FSW population in Kampala, April 2008 to April 2017.The cohort of FSW was established at a clinic located on Mengo hill, about 2 km from Kampala city center. This cohort (OBC_2_) recruited women from the city’s sex work hot spots, including clusters of bars, nightclubs, lodges and guesthouses. Both cohorts details have been previously described [8, 11, 14, 20, 24].

#### SiVET cohort

Two Simulated Vaccine Efficacy Trials were nested within the observational cohorts. SiVET_1_ (FF), in the FF communities, ran from June 2012 until April 2014. SiVET_2_ (FSW), in the FSW cohort, ran from August 2014 until April 2017. The SiVETs used a hepatitis B vaccine as a proxy for an HIV vaccine, with the aim of assessing acceptability of vaccination and retention in a future HIV vaccine trial environment. Cohort participants who had been enrolled for 3 to 18 months were consecutively screened for eligibility (Table 1) and enrolled until the required sample size was accrued. Those enrolled were administered a licensed Hepatitis B vaccine (ENGERIX-BTM GlaxoSmithKline Biologicals Rixensart, Belgium) following the standard schedule of 0, 1 and 6 months, under conditions that mimicked a possible HIV vaccine efficacy trial. In addition to the vaccination visits, participants in the SiVET cohort were followed up every 3 months for 12 months in line with the source observational cohort objective of determining HIV status every quarter.

**Table 1:** SiVETs and non-SiVETs cohorts’ participant eligibility criteria

SiVET cohorts	non-SiVET cohorts
**Inclusion** • At least 3 and no more than 18 months of follow up in the OBC_1_ or OBC_2_ • HIV-1 negative and willing to undergo HIV testing • Aged ≥18 years and ≤ 49 years • Able and willing to provide written informed consent • Able and willing to provide adequate locator information including physical address • Willing and able to return for follow-up clinic visits • Intending to reside in study area for at least one year **Females only** • Willing to undergo pregnancy testing • Not breastfeeding and no intent for pregnancy in the next one year • Willing to use effective contraception during the study and at least 3 months after the last vaccination	**Inclusion** • At least 3 months and no more than 18 months of follow up in OBC_1_ or OBC_2_ • Still in follow up in the OBCs • HIV-1 negative and willing to undergo HIV testing
**Exclusion**	**Exclusion**
• HIV positive • History of severe allergic reaction to any substance • An acute or chronic illness • Contraindication for Hepatitis B vaccine • Participation in another clinical trial • Hepatitis B exposure, as assessed by surface antigen (HBsAg) and core antibody (HBcAb) titers (only SiVET_2_) • Not willing to provide written consent	HIV positive

*SiVET*- Simulated Vaccine Efficacy Trial, *OBC-* Observational cohort

#### Non-SiVET cohort

**Fig. 1:**
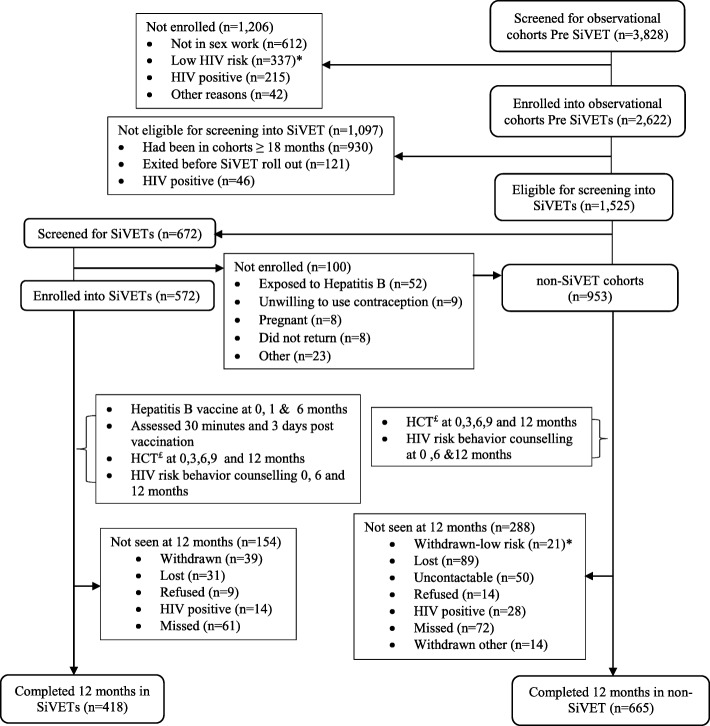
Study profile for participants screened and enrolled in observational cohorts before SiVET, in the non-SiVET and SiVET cohorts in the key populations, Uganda 2012–2017. *Low HIV risk defined as having protected sex with ≥one or new sexual partner, no history of STIs, non-use of illicit drugs and /or alcohol and not being away from home for ≥2 nights per/week, ^£^HIV counselling and Testing

Participants in OBC_1_ and OBC_2_ that screen failed SiVETs eligibility (Table 1), and those that were not screened because SIVET enrolment was complete, but fulfilled the criteria (Table 1) for continuing follow up in OBC_1_ (FF) and OBC_2_ (FSW), remained in follow up in the respective OBCs in the SiVET concurrent period, forming non-SiVET_1_ (FF) cohort in OBC_1_ and non-SiVET_2_ (FSW) cohort in OBC_2_. Participants in the non-SiVET cohort were followed up every 3 months for 12 months in the SiVET concurrent period (Fig. 1).

#### Retention strategies

##### SIVET cohorts

At the onset of the SiVET cohorts, each participant provided a cell phone number, and additionally a physical contact address and phone contact of a neighbor or someone who could easily reach them any time. This information was checked at each follow up clinic visit. Study field staff reminded participants of their next scheduled clinic visits using their cell phone at least 2 days before a scheduled clinic visit and visited their physical location the day after the scheduled visit if they did not attend. Participants who needed help to access the clinic were offered transport.

##### Non-SiVET cohorts

At the onset of the non-SIVET cohorts, each participant provided a cell phone contact number. This information was checked at each follow up clinic visit. When a participant missed a scheduled clinic visit the study field staff contacted him/her by cell phone and encouraged clinic attendance.

#### Definitions

##### Study completion

For the purpose of this paper, we defined study completion as a participant completing 12 months of follow up in the non-SiVET or SiVET cohorts concurrent period, or until HIV infection, or being withdrawn from a given cohort for any of the following reasons; reaction to hepatitis B vaccine, pregnancy (SiVETs only), being at low risk of HIV infection (non-SiVET_1_ only) and investigator discretion.

##### Lost to follow-up

This was defined as missing at least two sequential follow up clinic visits.

##### Dropout

This was defined as either lost to follow up, participant being uncontactable, refusal to continue or missing the 12-month study clinic visit.

##### Primary outcomes in this paper

This analysis compares the rate of dropout between SiVET and non-SiVET cohorts in the 12 months of SiVET concurrent period, reports the main reasons for dropping out, and investigates factors associated with dropout in each cohort.

### Statistical methods

The data collected in the non-SiVET cohorts were managed in MS Access, 2003 (Microsoft Corporation, Redmond, WA), while data from the SiVET cohorts were managed in OpenClinica 3.5 **(**Waltham, MA). We summarized participant characteristics using counts and percentage and compared them between non-SIVET and SiVET cohorts in the respective key population using chi-square tests. Participants who enrolled into either study and did not return for any follow up visit were given an arbitrary follow-up time of 1 week to allow inclusion in regression models. The dropout rate was estimated as the number of people who dropped out divided by the total person years of observation (PYO), expressed as a rate per 100 PYO. PYO were estimated as sum of the time from enrolment into SiVET to the date of SiVET completion or censoring. In the non-SiVET cohort, PYO were estimated as sum of time from the date SiVET began enrolment, ending on the date of the last SiVET participant clinic visit or date of censoring. Unadjusted rate ratios (uRR) and adjusted rate ratios (aRR) and their 95% confidence intervals (CI) were used to find factors associated with dropout by fitting Poisson regression models. Bivariable analysis was performed initially. Multivariable analysis was performed, including all variables which caused a change in the rate of more than 20%, except for sex and age which were included a priori.

## Results

### Screening and enrolment

In total 3828 participants were screened for possible enrolment into observational cohorts before SiVET rollout and 2622 (69%) were enrolled, Fig. 1. The main reasons for screen failure were non-involvement in sex work (*n* = 612), being at low risk for HIV infection (*n* = 337) and HIV infection (*n* = 215). At the time of introduction of the SiVET protocol, 1525 (58%) of the participants enrolled into observational cohorts before SiVETs were eligible for screening into SiVETs. The main reasons for ineligibility were having spent more than 18 months in observational cohorts (*n* = 930) and exiting observational cohorts before SiVET protocol roll out (*n* = 121), Fig. 1. Of the 1525 eligible for screening, 672 (44%) were consecutively screened and 572 (85%) of these enrolled into SiVETs (282 from FF and 290 from FSW). The main reason for screening but not enrolling into SiVETs was exposure to Hepatitis B (*n* = 52) (assessed as shown in Table 1). In total, 953 (283 from FF and 670 from FSW) participants were eligible for follow up in the non-SiVET cohorts in the SiVET concurrent period, Fig. 1.

### Baseline participant characteristics

#### FF

Compared to non-SiVET_1_ cohort, SiVET_1_ cohort had more men 73% vs 48% and more participants aged ≥35 years, 24% vs 14%, Table 2. Furthermore, non-SiVET_1_ cohort had more participants without any education 12% vs 6%, working in restaurant/bar/hair salon occupation 23% vs 8% and lived at the current location for 1 year or less 34% vs 17%, Table 2.

**Table 2:** Baseline characteristics of the participants in the non-SiVET and SiVET cohorts in FF and FSW populations in Uganda 2012–2017

Variables	FF(*N* = 565)			FSW(*N* = 960)		
	non-SiVET_1_ (*n* = 283)	SiVET_1_ (*n* = 282)	p -value	non-SiVET_2_ (*n* = 670)	SiVET_2_ (*n* = 290)	*p*-value
	Total (%)	Total (%)		Total (%)	Total (%)	
Sex			< 0.01			na
Male	137 (48)	205 (73)		na	na	
Female	146 (52)	77 (27)		670 (100)	290 (100)	
Age group (years)			0.01			< 0.01
18–24	127 (45)	88 (31)		304 (45)	85 (29)	
25–34	115 (41)	127 (45)		289 (43)	143 (49)	
35+	41 (14)	67 (24)		77 (12)	62 (22)	
Tribe			0.02			0.04
Baganda	114 (40)	128 (45)		295 (44)	153 (53)	
Banyankole	50 (18)	31 (11)		109 (16)	32 (11)	
Banyarwanda	69 (24)	54 (19)		40 (6)	20 (7)	
Other	50 (18)	69 (25)		226 (34)	85 (29)	
Education			0.04			< 0.01
None	35 (12)	19 (6)		272 (41)	16 (6)	
Primary	190 (67)	211 (75)		282 (42)	149 (51)	
Secondary+	58 (21)	52 (19)		116 (17)	125 (43)	
Marital status			0.17			0.01
Single never married	86 (30)	84 (30)		240 (36)	68 (24)	
Married	125 (44)	143 (51)		42 (6)	18 (6)	
Single ever married	72 (26)	55 (19)		388 (58)	204 (70)	
Religion			0.86			0.96
Christian	216 (76)	217 (77)		507 (76)	219 (76)	
Muslim	67 (24)	65 (23)		163 (24)	71 (24)	
Occupation			< 0.01			0.02
Fishing/fish related^b^	124 (44)	169 (60)		0 (0)	0 (0)	
Small scale business	59 (21)	73 (26)		17 (3)	11 (4)	
Work in restaurant/bar/hair salon	65 (23)	23 (8)		196 (29)	111 (38)	
Sex work	0 (0)	0 (0)		452 (67)	165 (57)	
Other^a^	35 (12)	17 (6)		5 (1)	3 (1)	
Duration lived at the current location (years)			< 0.01			< 0.01
0–1	96 (34)	48 (17)		222 (33)	51 (18)	
> 1	187 (66)	234 (83)		448 (67)	239 (82)	
Illicit drug use			0.29			0.79
No	254 (90)	245 (87)		132 (20)	55 (19)	
Yes	29 (10)	37 (13)		538 (80)	235 (81)	

*SiVET*- Simulated Vaccine Efficacy Trial, N-Total sample size, n-Sub study sample size, %-Percent, *na*- Not applicable, p-value compares SiVET to non-SiVET stratified by population

^a^Peasant farmer, house wife

^b^Drying fish, salting or smoking fish

#### FSW

Compared to the non-SiVET_2_ cohort, SiVET_2_ cohort had fewer participants aged ≥35 years, 12% vs 22%, Baganda tribe 44% vs 53% and those working in restaurant/bar/hair salon occupation 29% vs 38% Table 2. Additionally, the non-SiVET_2_ cohort had more participants without any education 41% vs 6%, single never married 36% vs 24% and those that lived at the current location for zero to one year 33% vs 18%, Table 2.

### Primary outcome (study dropout)

Among the 1525 participants, 326 (21%) dropped out of the cohorts. Of these 225/953 (24%) dropped out of the non-SiVET cohorts compared to 101/572 (18%), *p* = 0.01 in the SiVET cohorts.

### Dropout rates

Overall, 326 participants dropped out of cohorts in 1260 Person Years of Observation (PYO), a dropout rate of 25.9 /100 PYO, 95%CI:23.2–28.8. The dropout rate was higher in the non-SiVET cohorts 31.6, 95%CI: 27.8–36.1 compared to SiVET cohorts 18.4, 95%CI: 15.1–22.4, rate ratio (RR) =0.6, 95%CI: 0.5–0.7, Table 3. Stratifying the dropout rate by the study populations, it was still higher in the non-SiVET cohort compared to SiVET cohort in a given population but generally, the dropout rate was highest in the FSW population, Table 3.

**Table 3:** Dropout in non-SIVET and SIVET cohorts, FF and FSW populations, Uganda 2012–2017

Population	non-SiVET		SiVET		Rate ratio (95%CI)
	C/PYO	Rate-R_2_ (95%CI)	C/PYO	Rate-R_1_ (95%CI)	R_1_/ R_2_
FF	93/335	27.8 (22.7–34.0)	46/322	14.3 (10.7–19.0)	0.51 (0.37–0.71)
FSW	132/376	35.1 (29.6–41.6)	55/227	24.2 (18.6–31.6)	0.69 (0.50–0.96)
Overall	225/711	31.6 (27.8–36.1)	101/549	18.4 (15.1–22.4)	0.58 (0.46–0.73)

*FF*- Fisherfolk, *FSW* -Female sex worker, *C*- cases of dropout, *PYO-* person years of observation, *SiVET*- Simulated Vaccine Efficacy Trial

Similarly, comparing dropout rates by similar participant characteristics, the rates were generally higher in non-SiVET cohorts, except for participants that had lived at the current location for zero to 1 year in the SiVET_2_ cohort, Table 4.

**Table 4:** Dropout by participant characteristics in the non-SiVET and SiVET cohorts in FF and FSW populations, Uganda 2012–2017

Variables	FF(N = 565)					FSW(N = 960)				
	non-SiVET_1_ (n = 283)			SiVET_1_ (n = 282)		non-SiVET_2_ (n = 670)			SiVET_2_ (n = 290)	
	Total (%)	C/PYO	Rate	C/PYO	Rate	Total (%)	C/PYO	Rate	C/PYO	Rate
Sex										
Male	342 (60)	47/165	28.6	32/236	14.6	–	–	–	–	–
Female	223 (40)	46/170	27.0	14/86	16.2	960 (100)	132/376	35.1	55/227	24.2
Age group (years)										
18–24	215 (38)	45/150	30.0	16/103	15.6	389 (41)	66/162	40.9	22/64	34.4
25–34	242 (43)	35/135	26.0	25/144	17.3	432 (45)	52/163	31.9	27/111	24.3
35+	108 (19)	13/50	26.0	5/75	6.7	139 (14)	14/52	27.0	6/52	11.6
Tribe										
Baganda	242 (43)	43/128	33.7	22/146	15.0	448 (47)	58/177	32.8	30/118	25.4
Banyankole	81 (14)	17/62	27.3	4/36	11.2	141 (15)	25/60	41.7	5/26	19.6
Banyarwanda	123 (22)	20/86	23.1	6/62	9.7	60 (6)	8/19	42.1	5/16	31.5
Other	119 (21)	13/59	22.1	14/78	17.9	311 (32)	41/120	34.1	15/67	22.3
Education										
None	54 (9)	12/32	38.0	2/23	8.7	288 (30)	54/151	35.7	3/11	27.6
Primary	401 (71)	64/229	27.9	34/241	14.1	431 (45)	56/157	35.7	30/118	25.4
Secondary+	110 (20)	17/74	22.9	10/58	17.3	241 (25)	22/68	32.3	22/98	22.4
Marital status										
Single never married	170 (30)	33/97	34.1	19/99	19.2	308 (32)	52/132	39.5	11/58	18.9
Married	268 (47)	39/151	25.8	20/160	12.5	60 (6)	11/24	46.1	5/12	40.9
Single ever married	127 (23)	21/87	24.1	7/63	11.1	592 (62)	69/221	31.2	39/157	24.9
Religion										
Christian	433 (77)	78/244	32.0	35/247	14.1	726 (76)	106/277	38.3	42/170	24.7
Muslim	132 (23)	15/91	16.4	11/75	14.7	234 (24)	26/99	26.2	13/57	22.7
Occupation										
Fishing/fish related^b^	293 (52)	42/151	27.7	24/193	12.4	–	–	–		
Small scale business	132 (23)	14/73	19.1	15/82	18.1	28 (3)	3/13	23.0	1/9	10.8
Work in restaurant/bar/hair salon	88 (16)	31/61	50.5	5/27	18.6	307 (32)	35/118	29.6	19/87	21.7
Sex work	–	–	–	–	–	617 (64)	93/243	38.3	34/128	26.6
Other^a^	52 (9)	6/49	12.2	2/19	10.6	8 (1)	1/2	44.4	1/2	44.0
Duration lived at the current location (years)										
0–1	144 (25)	38/104	36.7	13/53	24.4	273 (28)	41/129	31.8	19/37	51.2
> 1	421 (75)	55/231	23.8	33/269	12.3	687 (72)	91/247	36.8	36/190	19.0
Illicit drug use										
No	499 (88)	84/298	28.1	38/278	13.7	187 (20)	31/63	49.5	8/43	18.6
Yes	66 (12)	9/37	24.6	8/44	18.1	773 (80)	101/314	32.2	47/184	25.5

*FF* -Fisherfolk*, FSW*- Female sex worker*, C*- cases of dropout*, PYO-* person years of observation*, SiVET*- Simulated Vaccine Efficacy Trial

^a^Peasant farmer, house wife

^b^Drying fish, salting or smoking fish

### Reasons for dropping out of cohorts

Of the 225 participants that dropped out of non-SiVET cohorts, 89 (40%) were lost to follow up other reasons are shown in Fig. 1. Similarly, of 101 participants that dropped out of the SiVET cohorts, 31 (31%) were lost to follow up, Fig. 1.

### Factors associated with dropout

#### FF

Factors independently associated with dropout in the non-SiVET_1_ cohort included sex [female: adjusted rate ratio (aRR) = 0.5, 95%CI: 0.3–0.9)], religion [Muslim: 0.4 (0.2–0.8)], occupation [work in restaurant/bar/hair salon: 3.1(1.3–7.4) compared to being engaged in small-scale business], other factors are shown in Table 5. In SiVET_1_ cohort_,_ only duration lived at the current location [> 1 year: 0.5 (0.3–0.9)] was independently associated with dropout.

**Table 5:** Unadjusted and adjusted factors associated with dropout in the non-SiVET and SiVET in FF and FSW, Uganda (2012–2017)

Variables	Fisherfolks (N = 565)				Female Sex Workers (N = 960)			
	non-SiVET_1_ (n = 283)		SiVET_1_ (n = 282)		non-SiVET_2_ (n = 670)		SiVET_2_ (n = 290)	
	uRR (95%CI)	aRR (95%CI)	uRR (95%CI)	aRR (95%CI)	uRR (95%CI)	aRR (95%CI)	uRR (95%CI	aRR (95%CI)
Sex								
Male	1	1	1	1	na	na	na	na
Female	0.9 (0.6–1.4)	**0.5 (0.3–0.9)**	1.2 (0.7–2.1)	0.8 (0.4–1.7)				
Age group (years)								
18–24	1	1	1	1	1	1	1	1
25–34	0.8 (0.6–1.4)	0.8 (0.5–1.3)	1.1 (0.7–1.9)	1.4 (0.8–2.4)	0.8 (0.5–1.2)	0.8 (0.5–1.4)	0.7 (0.4–1.2)	**0.6 (0.3–0.9)**
35+	0.8 (0.5–1.6)	0.8 (0.4–1.5)	0.4 (0.2–1.1)	0.5 (0.2–1.4)	0.7 (0.3–1.3)	0.7 (0.3–1.5)	0.3 (0.2–0.8)	**0.3 (0.1–0.7)**
Tribe								
Baganda	1		1		1		1	
Banyankole	0.8 (0.5–1.4)		0.7 (0.3–1.9)		1.3 (0.7–2.2)		0.8 (0.3–2.0)	
Banyarwanda	0.7 (0.4–1.2)		0.6 (0.3–1.5)		1.2 (0.5–3.1)		1.3 (0.5–3.2)	
Other	0.6 (0.4–1.2)		1.2 (0.7–2.1)		1.1 (0.7–1.7)		0.8 (0.5–1.6)	
Education								
None	1		1		1	1	1	
Primary	0.7 (0.4–1.4)		1.6 (0.5–5.7)		0.9 (0.6–1.6)	1.4 (0.8–2.2)	0.9 (0.3–3.1)	
Secondary+	0.6 (0.3–1.3)		2.0 (0.5–7.6)		0.9 (0.5–1.6)	1.2 (0.6–2.4)	0.8 (0.2–2.7)	
Marital status								
Single never married	1		1		1	1	1	1
Married	0.8 (0.5–1.2)		0.6 (0.4–1.1)		1.2 (0.5–2.6)	**2.2 (1.1–5.6)**	2.2 (0.7–6.4)	**4.0 (1.3–11.8)**
Single ever married	0.7 (0.4–1.2)		0.5 (0.3–1.3)		0.8 (0.5–1.2)	0.9 (0.6–1.6)	1.3 (0.7–2.5)	**2.2 (1.1–4.6)**
Religion								
Christian	1	**1**	1		1	**1**	1	
Muslim	0.5 (0.3–0.9)	**0.4 (0.2–0.8)**	1.1 (0.6–1.9)		0.7 (0.4–1.1)	**0.6 (0.3–0.9)**	0.9 (0.5–1.7)	
Occupation								
Small scale business	1	1	1		1		1	
Fishing/fish related^b^	1.5 (0.8–2.6)	1.5 (0.8–2.8)	0.7 (0.4–1.2)		–		–	
Work in restaurant/bar/hair salon	2.6 (1.4–5.0)	**3.1 (1.3–7.4)**	1.1 (0.5–2.4)		1.3 (0.4–4.6)		2.0 (0.3–14.4)	
Sex work	–	–	–		1.7 (0.5–5.8)		2.5 (0.4–17.1)	
Other^a^	0.6 (0.2–1.7)	0.8 (0.3–2.3)	0.6 (0.2–2.3)		1.9 (0.1–25.6)		4.1 (0.3–61.2)	
Duration lived at the current location (years)								
0–1	1	1	1	1	1	1	1	1
> 1	0.6 (0.4–0.9)	0.8 (0.5–1.3)	0.5 (0.3–0.9)	**0.5 (0.3–0.9)**	1.2 (0.8–1.8)	1.3 (0.8–2.1)	0.4 (0.2–0.6)	**0.4 (0.2–0.7)**
Illicit drug use								
No	1		1		1	1	1	
Yes	0.8 (0.4–1.7)		1.3 (0.7–2.5)		0.7 (0.4–1.1)	0.6 (0.3–0.9)	1.4 (0.7–2.9)	
Alcohol use								
Never	1		1		1		1	
Rarely	1.4 (0.9–2.1)		0.8 (0.5–1.3)		1.1 (0.6–2.1)		1.2 (0.6–2.4)	
Daily	0.9 (0.4–2.3)		0.4 (0.1–1.4)		1.4 (0.8–2.3)		1.8 (0.9–3.7)	
Sex while drunk								
Never	1	1	1	1	1		1	
Sometimes	0.9 (0.6–1.5)	**0.6 (0.3–0.9)**	1.2 (0.7–2.0)	1.3 (0.8–2.3)	1.5 (0.8–2.8)		1.3 (0.7–2.1)	
Frequently	1.1 (0.6–1.9)	0.7 (0.4–1.3)	0.3 (0.1–1.2)	0.4 (0.1–1.5)	1.3 (0.1–14.1)		2.3 (0.8–6.8)	
Genital discharge								
Yes	1		1		1		1	
No	0.8 (0.5–1.1)		1.2 (0.7–2.1)		0.8 (0.5–1.3)		0.7 (0.4–1.2)	
Genital ulcer/sores								
Yes	1		1		1		1	1
No	0.9 (0.6–1.3)		1.1 (0.7–1.9)		0.9 (0.2–1.6)		0.5 (0.3–0.9)	**0.5 (0.3–0.9)**
Number of sexual partners								
0–1	1	1	1	1	1	1	1	
2+	1.9 (1.3–2.9)	**1.7 (1.1–2.6)**	0.7 (0.4–1.3)	0.7 (0.4–1.3)	0.7 (0.4–1.5)	0.6 (0.3–1.4)	1.0 (0.4–2.5)	
New sexual partner								
No	1		1		1		1	
Yes	2.0 (1.1–3.9)		2.0 (1.2–3.6)		1.2 (0.5–2.8)		0.9 (0.5–1.9)	
Condom use with new sexual partner								
Never	1		1		1	1	1	
Sometimes	1.2 (0.7–1.9)		1.1 (0.6–1.9)		0.4 (0.2–0.7)	**0.4 (0.2–0.8)**	0.2 (0.1–0.9)	
Always	0.9 (0.6–1.6)		0.9 (0.4–2.1)		0.7 (0.5–1.2)	0.7 (0.4–1.2)	0.4 (0.1–1.9)	
Away^*c*^								
Yes	1	1	1	1	*–*		1	1
No	1.7 (1.1–2.5)	**2.0 (1.3–3.1)**	1.2 (0.7–2.0)	1.2 (0.7–2.1)			0.8 (0.5–1.4)	0.7 (0.4–1.3)

*uRR-* Unadjusted risk ratio*, aRR-* adjusted risk ratio*, CI*- Confidence interval*, %-Percent, SiVET-* Simulated Vaccine Efficacy Trial*, na-* not applicable*, bold-* statistically significant adjusted results

^a^Peasant farmer, house wife

^b^Drying fish, salting or smoking fish

^c^being away from home for ≥ 2 nights per/week

#### FSW

Factors independently associated with dropout in the non-SiVET_2_ cohort included religion [Muslim: 0.6 (0.3–0.9)], marital status [married: 2.2 (1.1–5.6) compared to single never married] and having sex under influence of alcohol [sometimes: 0.4 (0.2–0.8) compared to never]. In SiVET_2_ cohort, factors independently associated with dropout included age [25–34 years: 0.6 (0.3–0.9), 35 or more years: 0.3 (0.1–0.7) all compared to 18–24 years] and duration lived at the current location [> 1 year: 0.4 (0.2–0.7)], other factors are shown in Table 5.

## Discussion

We investigated how participant dropout rate from the Simulated HIV Vaccine Efficacy Trial (SiVET) differs from the observational cohort within which SiVET was nested. We compared participant dropout rate in SiVET to that in non-SiVET cohort adjusted to align over a set duration of time in two distinct key populations in Uganda. We found that the dropout rate in the SiVET cohort was nearly half that in the non-SiVET cohort. When stratified by the study population, the difference in dropout rate between SiVET and non-SiVET cohorts was generally similar though the dropout rates in either cohort were higher in the FSW population.

The results of this comparative analysis suggest that even when participants are drawn from the same population and followed up for the same duration of time, the selection criteria into efficacy trial and/or trial environment could cause a difference in trial dropout rate. Much as the observational cohorts were the recruitment source for the SiVETs, participants who joined SiVETs differed in significant ways from those who did not. SiVET recruited fewer females, young participants (< 25 years), not educated, working in restaurants/bar/hair salon, single and never married and those that had lived at the current location for a shorter duration (1 year or less). These participant characteristics have been previously associated with high attrition from observational cohorts in these [9, 11, 15] and other populations [25, 26]. Furthermore, these participant characteristics have also been previously associated with high risk of HIV acquisition in these populations [9, 15, 16, 19] and other HIV at-risk populations [27, 28].

SiVET cohorts’ lower dropout rate could also be attributable to the enhanced follow up procedures. SiVET cohorts’ participants were reminded of their next scheduled clinic visit at least 2 days in advance, and were picked by a trial staff on a motor cycle or vehicle if they needed help to access the clinic for their visits. Enhanced strategies to keep participants in follow up have been previously associated with high retention in follow up [29]. Furthermore, SiVET participants had four more clinic visits to complete trial procedures and adherence counselling. Regular study clinic visits have been associated with improved study outcomes and completion [30]. More clinic visits resulted in more HIV risk reduction counselling and other free Health care services in the SiVET cohort than non-SIVET cohort and a lower HIV incidence observed in this group [8].

The results suggest a number of factors were independently associated with dropout from the SiVET and non-SiVET cohorts including age, gender, occupation, marital status, duration lived at the current location, having sex while drunk, having multiple sexual partners, mobility, condom use with a new sexual partner, and genital sores/ulcer disease. These have previously been associated with more non-study completion in the key populations [6, 15, 20, 27, 31] and other populations [3, 32]. A surprising finding was that in FF and FSW non-SiVET cohorts, the rate of dropout among Muslims was statistically significantly lower than that among Christians. Though not statistically significant, a similar result was observed in the SiVET cohorts. While there is no clear explanation to this, Muslims have been indicated to be less likely to migrate [33] and over 90% of the dropouts were either uncontactable, lost to follow up or missed the last visit.

The commonest reasons for dropping out of the SiVET and non-SiVET cohorts were lost to follow up, being uncontactable and missing the last visit without giving investigators an opportunity to ascertain the actual reasons. Missing study visits has been associated with migration [11, 34]. Similarly, participants that migrate have been previously associated with increased risk of HIV infection because of high-risk sexual behaviours among those that move [11, 34]. SIVET cohort recruited more of the participants that had lived at the current location for more than 1 year. This could partly explain the lower drop out observed in SiVET cohort in this analysis and the lower HIV incidence in SiVET previously published [8]. Recruitment strategies aimed at avoiding participants that move could improve retention but screen out those likely to seroconvert. Therefore, researchers planning HIV vaccine efficacy trials in these populations need strategies aimed at retaining participants likely to be mobile, to minimize dropout rates and maximize HIV incidence.

Our study strengths included large sample size, two distinct key populations, aligning both SiVET and non-SiVET cohorts to the same duration of follow up in a concurrent period and participants being seen by the same study staff. Furthermore, participants were allowed a run in period of at least 3 months participation in the source cohort, mimicking a screening enrolment time lag in an actual HIV vaccine efficacy trial. Results from SiVETs suggest that with enhanced strategies, these populations could be enrolled and retained in HIV vaccine efficacy trials.

The limitations of this comparative analysis include, SiVET cohorts were likely to screen and enroll participants that came on time for their three - eighteen months visit in the source cohort. This could have filtered participants likely to come on time and completing study follow up visits beyond that seen in non-SiVET cohorts. This could have inadvertently decreased dropout in SiVETs compared to non-SiVET cohorts. Furthermore, although participants screened for HIV vaccine efficacy trials are required to have a run in period before actual recruitment, as it was done in SiVETs, it is unlikely that this will be up to three to eighteen months. The procedures in the SiVET and non-SiVET cohorts were conducted by the same study staff and were not blinded. This could have led to differential handling of follow up efforts to ensure that participants return for follow up. SiVET cohort participants were fully informed that the vaccine being administered has no effect on their risk of HIV infection but prevents against Hepatitis B infection. This could have led to participants continued attendance to enjoy enhanced health care services. Nonetheless, in an actual HIV vaccine efficacy trial, participants have to be informed that the vaccine being administered is not yet known to prevent against HIV infection. Even with these limitations, our comparative analysis gives a rare opportunity of estimating dropout rate in trials nested within source cohorts adjusting them to reflect the same duration of follow up in the same period and populations.

## Conclusion

In conclusions, HIV Vaccine Efficacy Trial’s inclusion-exclusion criteria and possibly some degree of bias in procedures, selected volunteers with characteristics different from those in the source population and not recruited. These in combination with trial environment and enhanced retention strategies reduced dropout rate from a trial in mobile populations. In HIV high-risk populations where no HIV prevention or simulation trials have been previously conducted to provide data for planning HIV vaccine efficacy trials, the dropout rate in observational cohort could be a useful tool. However, this rate might have to be decreased by 40% as observed in the SiVET cohorts in these key populations. Evidence further suggests that the decrease in dropout varied by population, 50% in FF and 30% in FSW. Entire results from these studies suggest that FF and FSW could be good populations for actual HIV vaccine efficacy trials because of the already known high HIV incidence and lower dropout rates in a trial setting as seen in the SiVETs.

## Data Availability

The MRC/UVRI and LSHTM Uganda Research Unit operates an open data access and has a data sharing policy accessible at https://www.mrcuganda.org/publications/data-sharing-policy. The policy summarizes the conditions under which data collected by the Unit can be made available to other bona fide researchers, the way in which such researchers can apply to have access to the data and how data will be made available if an application for data sharing is approved. Should any other researchers need to have access to the data from which this manuscript was generated, the processes to access the data are well laid out in the policy. The corresponding and other co-author emails have been provided and they could be contacted anytime for further clarifications and/or support to access the data.

## Abbreviations

AIDS: Acquired Immunodeficiency Syndrome
aRR: Adjusted Rate Ratio
CI: Confidence Interval
FF: Fisherfolk
FSW: Female Sex Worker
HIV: Human Immunodeficiency Virus
IAVI: International AIDS Vaccine Initiative
LSHTM: London School of Hygiene and Tropical Medicine
MA: Massachusetts
MRC: Medical Research Council
OBC: Observational Cohort
PYO: Person Years of Observation
RR: Rate Ratio
SiVET: Simulated Vaccine Efficacy Trial
UK: United Kingdom
uRR: Unadjusted Rate Ratio
USA: United States of America
USAID: United States Agency for International Development
UVRI: Uganda Virus Research Institute
WA: Washington
